# Machine learning prediction for mortality of patients diagnosed with COVID-19: a nationwide Korean cohort study

**DOI:** 10.1038/s41598-020-75767-2

**Published:** 2020-10-30

**Authors:** Chansik An, Hyunsun Lim, Dong-Wook Kim, Jung Hyun Chang, Yoon Jung Choi, Seong Woo Kim

**Affiliations:** 1 Research Institute, National Health Insurance Service Ilsan Hospital, Goyang, Korea; 2 Department of Big Data, National Health Insurance Service, Wonju, Korea; 3 Department of Otolaryngology-Head and Neck Surgery, National Health Insurance Service Ilsan Hospital, Goyang, Korea; 4 Present Address: Department of Pathology, Yongin Severance Hospital, Yonsei University College of Medicine, Yongin, Korea; 5 Department of Physical Medicine and Rehabilitation, National Health Insurance Service Ilsan Hospital, Goyang, Korea

**Keywords:** Viral infection, Machine learning

## Abstract

The rapid spread of COVID-19 has resulted in the shortage of medical resources, which necessitates accurate prognosis prediction to triage patients effectively. This study used the nationwide cohort of South Korea to develop a machine learning model to predict prognosis based on sociodemographic and medical information. Of 10,237 COVID-19 patients, 228 (2.2%) died, 7772 (75.9%) recovered, and 2237 (21.9%) were still in isolation or being treated at the last follow-up (April 16, 2020). The Cox proportional hazards regression analysis revealed that age > 70, male sex, moderate or severe disability, the presence of symptoms, nursing home residence, and comorbidities of diabetes mellitus (DM), chronic lung disease, or asthma were significantly associated with increased risk of mortality (*p* ≤ 0.047). For machine learning, the least absolute shrinkage and selection operator (LASSO), linear support vector machine (SVM), SVM with radial basis function kernel, random forest (RF), and k-nearest neighbors were tested. In prediction of mortality, LASSO and linear SVM demonstrated high sensitivities (90.7% [95% confidence interval: 83.3, 97.3] and 92.0% [85.9, 98.1], respectively) and specificities (91.4% [90.3, 92.5] and 91.8%, [90.7, 92.9], respectively) while maintaining high specificities > 90%, as well as high area under the receiver operating characteristics curves (0.963 [0.946, 0.979] and 0.962 [0.945, 0.979], respectively). The most significant predictors for LASSO included old age and preexisting DM or cancer; for RF they were old age, infection route (cluster infection or infection from personal contact), and underlying hypertension. The proposed prediction model may be helpful for the quick triage of patients without having to wait for the results of additional tests such as laboratory or radiologic studies, during a pandemic when limited medical resources must be wisely allocated without hesitation.

## Introduction

A pneumonia of unknown cause detected in Wuhan, China was first reported to the World Health Organization (WHO) on December 31, 2019. A few weeks later, it was found to be caused by a novel coronavirus^1^. On February 11, 2020, the WHO formally named the causative coronavirus the severe acute respiratory syndrome coronavirus 2 (SARS-CoV-2) and the disease caused by the virus, the coronavirus disease 2019 (COVID-19). COVID-19 is the third known zoonotic coronavirus disease after SARS and the Middle East Respiratory Syndrome (MERS)^2^. After the onset of its rapid spread worldwide, the WHO declared the COVID-19 outbreak a global pandemic on March 11, 2020.

COVID-19 has a higher mortality rate (3.8% according to the WHO as of August 2020) than influenza and spreads more rapidly over much wider areas than prior coronavirus diseases. COVID-19 has already claimed far more lives than its predecessors (813 deaths for SARS and 858 deaths for MERS)^3,4^. As of October 05, 2020, COVID-19 had infected over 35 million people with the worldwide death toll exceeding 1,040,000^5^.

Because of the rapid spread of the virus, there has been a sharp increase in the demand for medical resources required to support infected people. Despite the desperate efforts to contain the disease and slow down its spread, many countries have been suffering from the shortage of hospital beds and critical care equipment for the timely treatment of ill patients^6–8^. Therefore, in addition to efficient diagnosis and treatment, accurate prognosis prediction is necessary to reduce the strain on healthcare systems and provide the best possible care for patients. When allocating limited medical resources, prediction models that estimate the risk of a poor outcome in an infected individual based on pre-diagnosis information could help to effectively triage patients.

All Koreans are mandated to enroll in national health insurance, except for the population in the lowest income bracket, who is funded by taxes and covered by Medicaid. Consequently, information regarding the sociodemographic characteristics and history of medical service use of virtually all Koreans is available in the database where currently information regarding COVID-19 patients is also periodically updated.

The purpose of this study was to develop and validate machine learning models that predict the prognosis of COVID-19 patients based on sociodemographic information, infection route, and medical status and history, for the nationwide cohort of South Korea.

## Results

### Baseline characteristics

The patient characteristics are summarized in Table 1. The mean (± standard deviation [SD]) age was 44.97 (± 19.79) years; patients who died of COVID-19 were significantly older than those who recovered, with the mean (± SD) age being 78.17 (± 10.96) years and 43.06 (± 18.32) years, respectively. Women comprised 60.1% of the study population. Approximately 38.5% of the patients were symptomatic at the time of diagnosis. A majority of the infections (58.1%) were cluster infection, and 11.3% of the patients were nursing home residents. Of the 10,237 patients, 3147 (30.7%) had one or more underlying medical conditions; the four most common conditions were hypertension (18.2%), hyperlipidemia (18.0%), chronic lung disease or asthma (10.5%), and diabetes mellitus (DM) (10.0%).

**Table 1:** Baseline characteristics.

		Mortality (N = 228)	Survived		Total (N = 10,237)
			Recovered (N = 7772)	Undetermined* (N = 2237)	
Age (years)	< 40	1 (0.4%)	3520 (45.3%)	907 (40.5%)	4428 (43.3%)
	40–50	3 (1.3%)	1093 (14.1%)	231 (10.3%)	1327 (13.0%)
	50–60	12 (5.3%)	1529 (19.7%)	338 (15.1%)	1879 (18.4%)
	60–70	30 (13.2%)	1023 (13.2%)	332 (14.8%)	1385 (13.5%)
	70–80	65 (28.5%)	429 (5.5%)	204 (9.1%)	698 (6.8%)
	> 80	117 (51.3%)	178(2.3%)	225(10.1%)	520 (5.1%)
Sex	Female	121 (53.1%)	4790 (61.6%)	1252 (56.0%)	6149 (60.1%)
	Male	107 (46.9%)	2982 (38.4%)	985 (44.0%)	4088 (39.9%)
Income level†	Medicaid	42 (18.4%)	569 (7.3%)	260 (11.6%)	871 (8.5%)
	< 25%	39 (17.1%)	2000 (25.7%)	453 (20.3%)	2492 (24.3%)
	25–50%	29 (12.7%)	1437 (18.5%)	334 (14.9%)	1800 (17.6%)
	50–75%	35 (15.4%)	1675 (21.6%)	436 (19.5%)	2146 (21.0%)
	> 75%	82 (36.0%)	2032 (26.1%)	747 (33.4%)	2861 (27.9%)
Residence	Suburban/rural	2 (0.9%)	240 (3.1%)	419 (18.7%)	661 (6.5%)
	Urban	149 (65.4%)	5657 (72.8%)	1018 (45.5%)	6824 (66.7%)
	Metropolitan	77 (33.8%)	1875 (24.1%)	800 (35.8%)	2752 (26.9%)
Household type	Others	161 (70.6%)	7503 (96.5%)	2044 (91.4%)	9708 (94.8%)
	Seniors (> 65 y) living alone	67 (29.4%)	269 (3.5%)	193 (8.6%)	529 (5.2%)
Disability	None	166 (72.8%)	7352 (94.6%)	1959 (87.6%)	9477 (92.6%)
	Mild	40 (17.5%)	301 (3.9%)	175 (7.8%)	516 (5.0%)
	Moderate or severe	22 (9.6%)	119 (1.5%)	103 (4.6%)	244 (2.4%)
Symptom	Absent	133 (58.3%)	4370 (56.2%)	1791 (80.1%)	6294 (61.5%)
	Present	95 (41.7%)	3402 (43.8%)	446 (19.9%)	3943 (38.5%)
Infection route	Personal contact	16 (7.0%)	1043 (13.4%)	250 (11.2%)	1309 (12.8%)
	Cluster infection	26 (11.4%)	5379 (69.2%)	542 (24.2%)	5947 (58.1%)
	Nursing home	127 (55.7%)	443 (5.7%)	584 (26.1%)	1154 (11.3%)
	From abroad	0 (0.0%)	188 (2.4%)	710 (31.7%)	898 (8.8%)
	Unclassified	59 (25.9%)	719 (9.3%)	151 (6.8%)	929 (9.1%)
Underlying medical condition‡	None	26 (11.4%)	5733 (73.8%)	1331 (59.5%)	7090 (69.3%)
	Hypertension	165 (72.4%)	1154 (14.8%)	545 (24.4%)	1864 (18.2%)
	Diabetes mellitus	107 (46.9%)	580 (7.5%)	334 (14.9%)	1021 (10.0%)
	Hyperlipidemia	112 (49.1%)	1252 (16.1%)	479 (21.4%)	1843 (18.0%)
	Cardiovascular disease	70 (30.7%)	280 (3.6%)	161 (7.2%)	511 (5.0%)
	Cerebrovascular disease	4 (1.8%)	5 (0.1%)	18 (0.8%)	27 (0.3%)
	Cancer	9 (3.9%)	40 (0.5%)	27 (1.2%)	76 (0.7%)
	Chronic lung disease or Asthma	93 (40.8%)	730 (9.4%)	257 (11.5%)	1080 (10.5%)
	Chronic renal disease	13 (5.7%)	42 (0.5%)	23 (1.0%)	78 (0.8%)
	Mental illness	58 (25.4%)	126 (1.6%)	313 (14.0%)	497 (4.9%)
	Chronic liver disease	10 (4.4%)	157 (2.0%)	64 (2.9%)	231 (2.3%)
Medication‡	ACE inhibitor	5 (2.2%)	30 (0.4%)	13 (0.6%)	48 (0.5%)
	AR blocker	62 (27.2%)	636 (8.2%)	224(10.0%)	922 (9.0%)
	Beta blocker	29 (12.7%)	189 (2.4%)	89 (4.0%)	307 (3.0%)
	Calcium channel blocker	59 (25.9%)	529 (6.8%)	209 (9.3%)	797 (7.8%)
	Loop diuretics	14 (6.1%)	21 (0.3%)	21 (0.9%)	56 (0.5%)
	Acarbose	2 (0.9%)	3 (0.0%)	2 (0.1%)	7 (0.1%)
	Sulfonylurea	22 (9.6%)	125 (1.6%)	65 (2.9%)	212 (2.1%)
	Metformin	45 (19.7%)	261 (3.4%)	117 (5.2%)	423 (4.1%)
	DDP-4	26 (11.4%)	141 (1.8%)	62 (2.8%)	229 (2.2%)
	Fenofibrate	4 (1.8%)	44 (0.6%)	15 (0.7%)	63 (0.6%)
	Statin	69 (30.3%)	742 (9.5%)	263 (11.8%)	1074 (10.5%)
	NSAID	12 (5.3%)	64 (0.8%)	29 (1.3%)	105 (1.0%)
	Aspirin	57 (25.0%)	305 (3.9%)	136 (6.1%)	498 (4.9%)

Recovered or undetermined cases were censored at the date of recovery or the date of last follow-up (April 16, 2020), respectively.

*ACE* angiotensin-converting enzyme, *AR* angiotensin receptor, *NSAID* non- steroidal anti-inflammatory drug.

*In isolation or under treatment.

^†^67 patients with missing values were excluded.

^‡^Some patients had more than one medical condition or medication.

### Mortality from COVID-19

**Figure 1:**
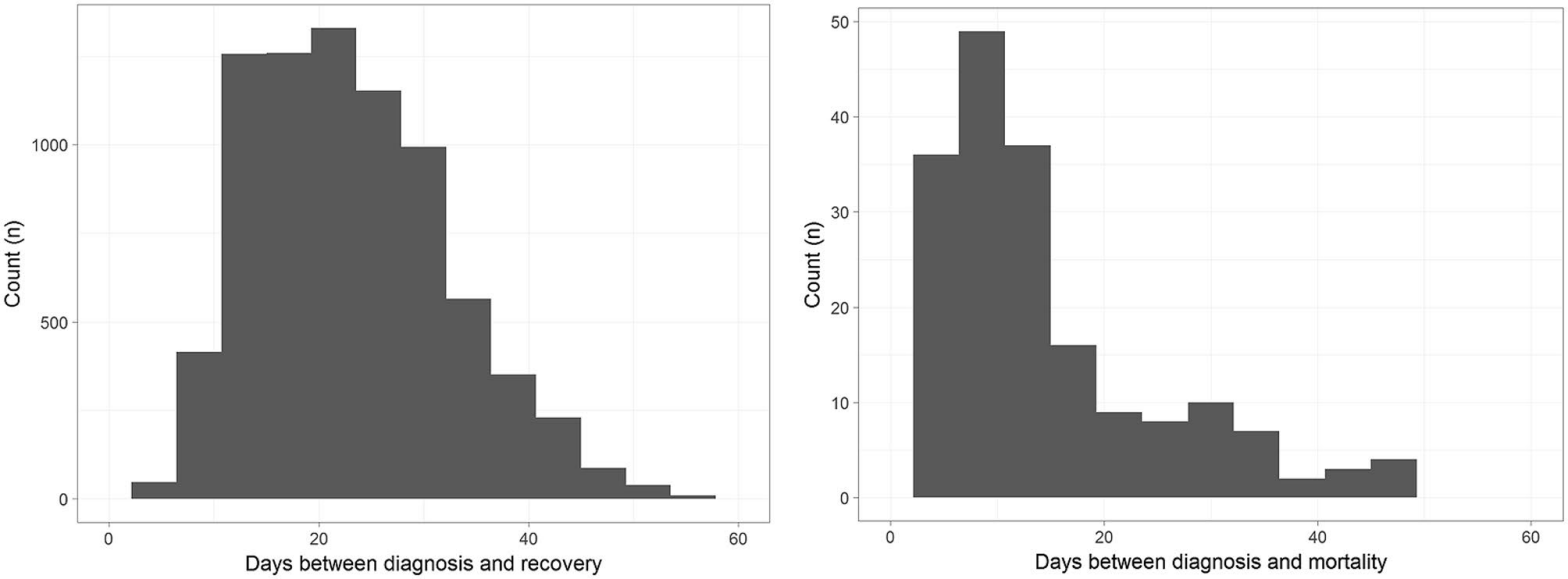
Histogram illustrating the distribution of the time interval between diagnosis and recovery (**A**) or mortality (**B**).

**Figure 2:**
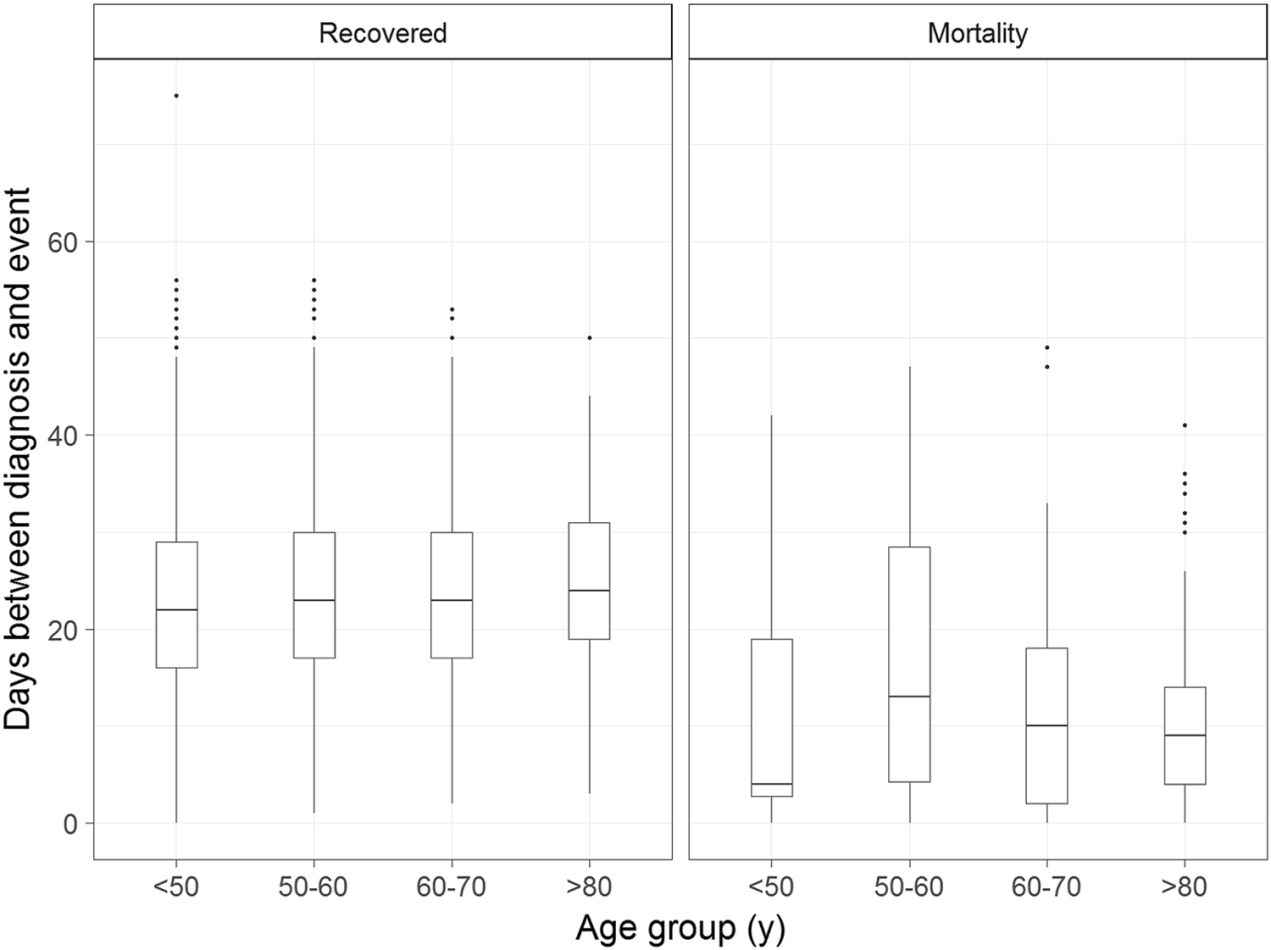
Box plot illustrating the time interval between diagnosis and recovery or mortality according to the age group.

A total of 10,237 patients were diagnosed with COVID-19 between January 23, 2020 and April 2, 2020 and were followed up until April 16, 2020. The case fatality ratio was 2.85% (228 deaths and 7772 recoveries). The median interval between diagnosis and mortality was 9 days (range 0–49 days), and 22 days (range 0–75 days) between diagnosis and recovery (Fig. 1). No significant difference in the interval between diagnosis and mortality or recovery was found among different age groups (*p* > 0.231) (Fig. 2).

### Factors associated with mortality

#### Cox proportional hazards model

In the multivariable analysis with medication excluded (Table 2), age > 70, male sex, moderate or severe disability, the presence of symptoms, infection at a nursing home, DM, and chronic lung disease or asthma were significantly associated with increased risk of mortality (*p* ≤ 0.047), whereas age < 40 and cluster infection or infection from personal contact or visit were associated with decreased risk (*p* ≤ 0.007). In the multivariable analysis with underlying disease excluded (Table 3), the use of loop diuretics or acarbose was significantly associated with increased risk of mortality (*p* ≤ 0.018).

**Table 2:** Results of Cox proportional hazards regression without medication.

		Univariable		Multivariable	
		HR (95% CI)	*p*-value	HR (95% CI)	*p*-value
Age (years)	< 40	0.04 (0.01, 0.28)	0.001	0.06 (0.01, 0.46)	0.007
	40–50	0.35 (0.10, 1.25)	0.107	0.47 (0.13, 1.68)	0.246
	50–60	Reference			
	60–70	3.12 (1.58, 6.17)	0.001	1.97 (0.99, 3.93)	0.054
	70–80	13.49 (7.23, 25.17)	< .0001	7.31 (3.77, 14.16)	< .0001
	> 80	40.49 (22.30, 73.50)	< .0001	17.46 (9.01, 33.85)	< .0001
Sex	Female	Reference			
	Male	1.86 (1.42, 2.44)	< .0001	2.37 (1.78, 3.15)	< .0001
Income level	Medicaid	3.35 (2.12, 5.29)	< .0001	1.34 (0.82, 2.19)	0.250
	< 25%	Reference			
	25–50%	1.07 (0.65, 1.77)	0.781	1.35 (0.81, 2.26)	0.247
	50–75%	1.18 (0.74, 1.88)	0.492	1.15 (0.71, 1.85)	0.566
	> 75%	1.89 (1.26, 2.83)	0.002	1.05 (0.69, 1.60)	0.814
Residence	Suburban/rural	Reference			
	Urban	0.72 (0.54, 0.96)	0.023	1.29 (0.94, 1.77)	0.119
	Metropolitan	0.13 (0.03, 0.53)	0.004	0.82 (0.20, 3.40)	0.784
Household type	Others	reference			
	Seniors (> 65 y) living alone	8.33 (6.21, 11.2)	< .0001	1.06 (0.76, 1.48)	0.717
Disability	None	Reference			
	Mild	4.76 (3.32, 6.82)	< .0001	0.98 (0.67, 1.42)	0.911
	Moderate or severe	6.19 (3.96, 9.68)	< .0001	1.63 (1.01, 2.63)	0.047
Symptom	Absent	reference			
	Present	1.08 (0.82, 1.42)	0.591	2.29 (1.70, 3.09)	< .0001
Infection route	Unclassified	Reference			
	Large clusters	0.08 (0.05, 0.12)	< .0001	0.31 (0.19, 0.52)	< .0001
	Nursing home	2.42 (1.73, 3.38)	< .0001	1.68 (1.10, 2.56)	0.017
	Personal contact	0.22 (0.12, 0.39)	< .0001	0.24 (0.13, 0.43)	< .0001
Underlying medical condition	None	Reference			
	Hypertension	12.18 (9.02, 16.46)	< .0001	1.22 (0.87, 1.73)	0.254
	Diabetes mellitus	8.30 (6.33, 10.89)	< .0001	1.75 (1.29, 2.36)	0.001
	Hyperlipidemia	4.27 (3.26, 5.60 )	< .0001	0.89 (0.66, 1.20)	0.446
	Cardiovascular disease	8.48 (6.29, 11.42)	< .0001	1.23 (0.89, 1.70)	0.220
	Cerebrovascular disease	8.53 (3.17, 22.96)	< .0001	0.88 (0.32, 2.44)	0.801
	Cancer	5.06 (2.38, 10.75)	< .0001	1.64 (0.75, 3.60)	0.216
	Chronic lung disease or Asthma	5.54 (4.20, 7.31)	< .0001	1.83 (1.37, 2.46)	< .0001
	Chronic renal disease	9.37 (5.35, 16.43)	< .0001	1.47 (0.80, 2.69)	0.215
	Mental illness	8.49 (6.22, 11.57)	< .0001	1.01 (0.69, 1.49)	0.948
	Chronic liver disease	1.74 (0.86, 3.52)	0.126	0.76 (0.37, 1.57)	0.462

*HR* hazard ratio, *CI* confidence interval; 0651.

**Table 3:** Results of Cox proportional hazards regression for medication.

	Univariable		Multivariable*	
	HR (95% CI)	*p*-value	HR (95% CI)	*p*-value
ACE inhibitor	4.30 (1.60, 11.56)	0.004	0.58 (0.20, 1.68)	0.314
AR blocker	3.58 (2.63, 4.87)	< .0001	0.93 (0.65, 1.32)	0.668
Beta blocker	4.77 (3.16, 7.19)	< .0001	1.18 (0.73, 1.88)	0.502
Calcium channel blocker	3.81 (2.77, 5.24)	< .0001	1.03 (0.72, 1.48)	0.875
Loop diuretics	14.64 (8.35, 25.67)	< .0001	2.17 (1.14, 4.11)	0.018
Acarbose	15.45 (3.84, 62.20)	0.001	8.36 (1.89, 36.93)	0.005
Sulfonylurea	5.43 (3.46, 8.53)	< .0001	1.12 (0.66, 1.93)	0.671
Metformin	5.82 (4.13, 8.18)	< .0001	1.41 (0.86, 2.33)	0.179
DDP-4 inhibitor	5.84 (3.82, 8.93)	< .0001	1.29 (0.75, 2.21)	0.358
Fenofibrate	3.47 (2.57, 4.68)	< .0001	0.87 (0.59, 1.28)	0.470
Statin	3.23 (1.20, 8.68)	0.020	1.23 (0.44, 3.44)	0.688
NSAID	5.11 (2.71, 9.65)	< .0001	1.31 (0.68, 2.53)	0.424
Aspirin	6.58 (4.80, 9.02)	< .0001	1.19 (0.79, 1.79)	0.397

*Adjusted for age, sex, income level, residence, household type, disability, symptom, and infection route.

*HR* hazard ratio, *CI* confidence interval, *ACE* angiotensin-converting enzyme, *AR* angiotensin receptor, *DDP-4* dipeptidyl peptidase-4, *NSAID* non- steroidal anti-inflammatory drug.

#### Variable importance from machine learning

**Figure 3:**
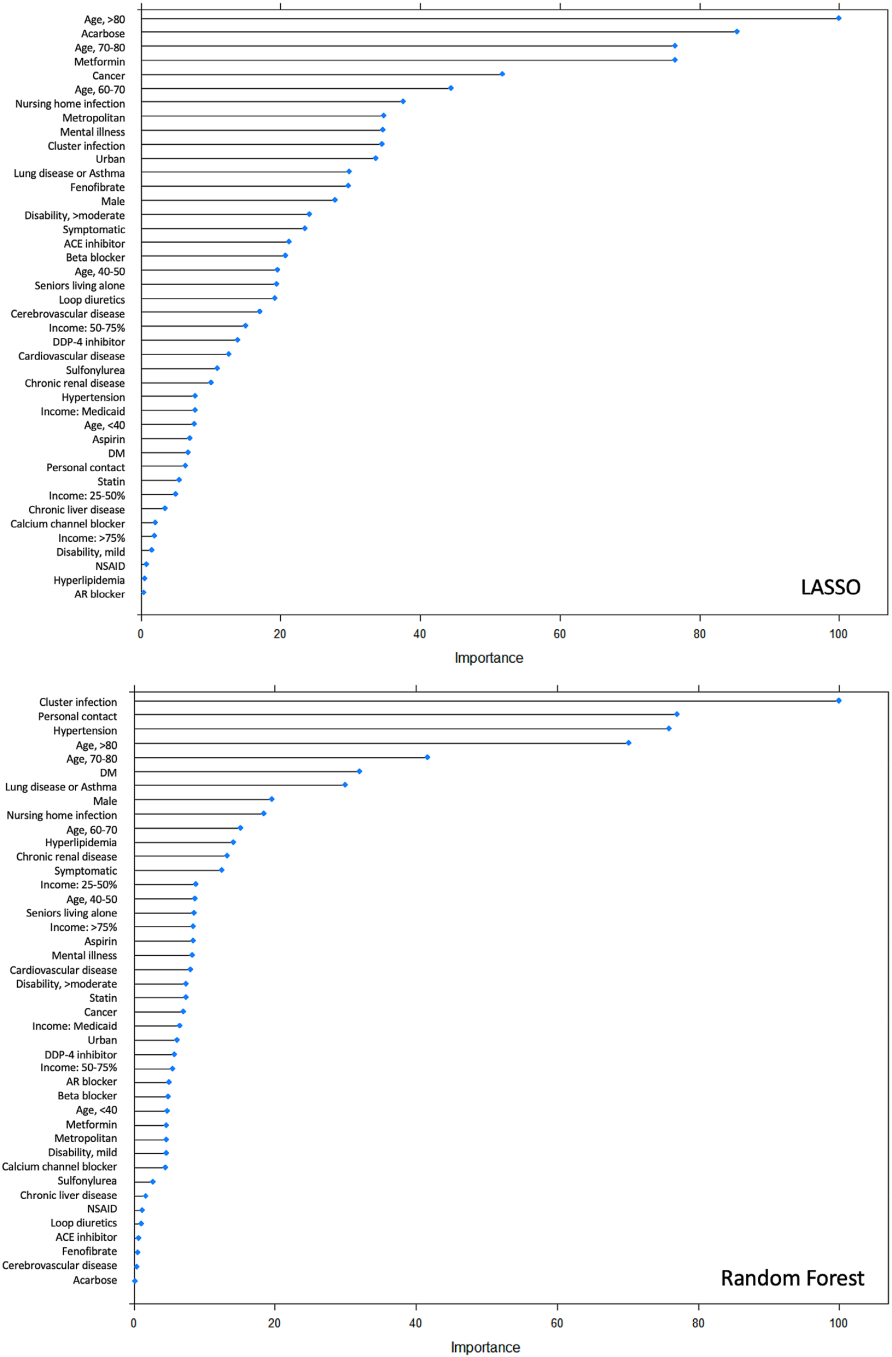
Variable importance in prediction of mortality from COVID-19 by LASSO (**A**) and Random Forest (**B**).

In predicting the final outcome (i.e., mortality vs. recovery), the five most significant predictors for the least absolute shrinkage and selection operator (LASSO) were age > 80, taking of acarbose, age > 70, taking of metformin, and underlying cancer in order of significance; for random forest (RF) they were cluster infection, infection from personal contact or visit, underlying hypertension, and age > 80 (Fig. 3). In predicting early mortality, similar patterns were observed (Supplementary Figs. 1 and 2). Overall, in addition to old age, LASSO focused on DM medication and cancer whereas RF relied on the infection route and hypertension.

### Performance of machine learning

The performances of the machine learning models in the final testing are summarized in Table 4. The optimized hyperparameters can be found in Supplementary Table 1. LASSO and linear support vector machine (SVM) showed high areas under the receiver operating characteristic curves (AUCs) (> 0.9), high balanced accuracies (> 91% for final mortality and > 86% for 14- or 30-day mortality), and sensitivities (> 90% for final mortality and > 83% for 14- or 30-day mortality) without compromising specificities (approximately 90%). However, the sensitivities of RF and radial basis function (RBF)-SVM were < 50%, ranging between 10.2% and 42.7% despite their high AUCs and specificities. K-nearest neighbor (KNN) showed the lowest AUCs and intermediate sensitivities and balanced accuracies. Regardless of the machine learning model, negative predictive values (NPVs) were high (> 97%), and positive predictive values (PPVs) were low (13.0–44.4%). All the models displayed have higher performances in long-term prediction than in short-term prediction.

**Table 4:** Final performance of machine learning models in prediction of mortality from COVID-19 in the test set.

Classifier	AUC	TP/FP/FN/TN	Sensitivity	Specificity	PPV	NPV	Balanced accuracy
**Mortality vs. recovery (with undetermined cases excluded)**							
LASSO	0.963 (0.946, 0.979)	68/208/7/2217	90.7% (83.3, 97.3)	91.4% (90.3, 92.5)	24.6% (19.7, 30.2)	99.7% (99.4, 99.9)	91.1% (86.0, 94.3)
Linear SVM	0.962 (0.945, 0.979)	69/199/6/2226	92.0% (85.9, 98.1)	91.8% (90.7, 92.9)	25.7% (20.6, 31.4)	99.7% (99.4, 99.9)	91.9% (87.0, 95.0)
RBF-SVM	0.958 (0.945, 0.971)	32/53/43/2372	42.7% (31.5, 53.9)	97.8% (91.9, 1)	37.6% (27.4, 48.8)	98.2% (97.6, 98.7)	70.2% (64.2, 76.5)
RF	0.958 (0.936, 0.981)	24/30/51/2395	32.0% (21.6, 42.8)	98.8% (98.4, 99.3)	44.4% (30.9, 58.6)	97.9% (97.3, 98.4)	65.4% (60.0, 71.5)
KNN	0.897 (0.856, 0.937)	61/255/14/2170	81.3% (73.1, 90.6)	89.5% (88.3, 90.7)	19.3% (15.1, 24.1)	99.4% (98.9, 99.6)	85.4% (79.5, 90.1)
**Mortality vs. survival within 14 days after diagnosis**							
LASSO	0.944 (0.921, 0.967)	44/293/9/2871	83.0% (72.9, 91.5)	90.7% (89.7, 91.9)	13.1% (9.6, 17.1)	99.7% (99.4, 99.9)	86.8% (80.0, 91.8)
Linear SVM	0.941 (0.914, 0.967)	45/303/8/2861	84.9% (75.3, 93.0)	90.4% (89.4, 91.6)	12.9% (9.6, 16.9)	99.7% (99.5, 99.9)	87.7% (80.8, 92.3)
RBF-SVM	0.919 (0.883, 0.955)	6/18/47/3146	11.3% (0.3, 18.0)	99.4% (99.1, 99.7)	25% (9.8, 46.7)	98.5% (98.0, 98.9)	55.4% (51.7, 61.4)
RF	0.925 (0.893, 0.958)	12/41/41/3123	22.6% (11.3, 32.1)	98.7% (98.3, 99.2)	22.6% (12.3, 36.2)	98.7% (98.2, 99.1)	60.7% (55.2, 67.7)
KNN	0.772 (0.705, 0.839)	32/205/21/2959	60.4% (47.2, 71.4)	93.5% (92.6, 04.4)	13.5% (9.4, 18.5)	99.3% (98.9, 99.6)	77.0% (69.3, 84.0)
**Mortality vs. survival within 30 days after diagnosis**							
LASSO	0.953 (0.937, 0.969)	57/309/7/2791	89.1% (81.4, 96.2)	90.0% (88.9, 91.2)	15.6% (12.0, 19.7)	99.7% (99.5, 99.9)	89.5% (83.8, 93.3)
Linear SVM	0.948 (0.928, 0.968)	55/324/9/2776	85.9% (77.3, 93.8)	89.5% (88.4, 90.7)	14.5% (11.1, 18.5)	99.7% (99.4, 99.9)	87.7% (81.7, 92.0)
RBF-SVM	0.915 (0.885, 0.944)	14/34/50/3066	21.9% (11.7, 31.3)	98.9% (98.5, 99.3)	29.2% (17.0, 44.1)	98.4% (97.9, 98.8)	60.4% (55.5, 66.6)
RF	0.946 (0.930, 0.963)	9/21/55/3079	14.1% (6.2, 21.9)	99.3% (98.9, 99.6)	30.0% (14.7, 49.4)	98.2% (97.7, 98.7)	56.7% (52.8, 62.3)
KNN	0.750 (0.687, 0.813)	37/247/27/2853	57.8% (44.9, 68.3)	92.0% (91.0, 93.0)	13.0% (9.3, 17.5)	99.1% (98.6, 99.4)	74.9% (67.9, 81.5)

Values in parentheses are 95% confidence intervals.

*AUC* area under the receiver operating characteristic curve, *TP* true positive, *FP* false positive, *FN* false negative, *TN* true negative, *PPV* positive predictive value, *NPV* negative predictive value, *LASSO* least absolute shrinkage and selection operator, *SVM* support vector machine, *RBF* radial basis function kernel, *RF* random forest, *KNN* k-nearest neighbors.

## Discussion

Our results demonstrate that machine learning models utilizing sociodemographic characteristics and medical history can accurately predict the prognosis of COVID-19 patients after diagnosis; the models predict not only the final outcome (i.e., mortality vs. recovery) but also early mortality (i.e., 14- or 30-day mortality). The proposed prediction model aims at the quick triage of patients without having to wait for the results of additional tests such as laboratory or radiologic studies, during a pandemic when limited medical resources must be wisely allocated without hesitation.

Machine learning is focused on achieving high predictive accuracy without much focus on explaining how the accuracy is achieved. We presented the result of the Cox proportional hazard regression and variable importance as complements, showing how importantly input variables were used by LASSO and RF. In line with previous studies^9–20^, old age, male gender, and the presence of symptoms or underlying medical conditions were significantly associated with worse prognosis. We additionally found that moderate or severe disability and infection route were independently associated with prognosis.

Almost all previous studies reported that old age was a strong prognostic factor, and this was also confirmed by our results. However, the time to recovery or mortality did not differ by age in our study population.

Previously reported underlying medical conditions associated with poor prognosis include hypertension^10,12,13,15,17,18^, DM^10,12,14,15^, lung disease including chronic obstructive lung disease and asthma^11–13^, cardiovascular disease^10,11,13–15^, cancer^21,22^, and chronic renal disease^12,18^. In our study, DM (from Cox and LASSO), chronic lung disease or asthma (from Cox regression), cancer (from LASSO), and hypertension (from RF) were significant predictors. LASSO paid more attention to DM medication than the disease itself, which may have been because patients taking medication were more likely to have had DM longer than those not taking it. The insignificance of the other medical conditions, such as cardiovascular disease or chronic renal disease in our study, may be due to a different study population, the broadness of our operational definition of the diseases, and correlation with other strong predictive factors.

We also performed multivariable Cox regression to identify drugs associated with increased or decreased risk of mortality and found that the use of loop diuretics or acarbose was an independent risk factor. However, these results need to be interpreted cautiously. There may have been other confounding factors among the patients who consume this medication that we were unable to sufficiently adjust for. Loop diuretics are recommended to be considered in patients with congestive heart failure or advanced chronic renal disease^23^. Alpha-glucosidase inhibitors including acarbose are often used with a basal insulin regimen when basal insulin treatment alone did not result in glycemic control, especially postprandial glucose level in Asians^24^. Thus, the poor outcome in the patients taking the medications may have been due to the fact that they had have a longer duration of more comorbidities, not due to the direct drug effects.

Our main interest for the medication analysis was the angiotensin converting enzyme (ACE) inhibitors and angiotensin receptor (AR) blockers. There have been concerns regarding a potential harmful effect caused by ACE inhibitors and AR blockers in COVID-19 patients^25^. In our study, however, the use of ACE inhibitors or AR blockers was not significantly associated with mortality from COVID-19, in agreement with a recent large-scale study^11^.

Recent discussions pointed out the potential beneficial or harmful effects of other commonly prescribed drugs including antidiabetic drugs, statin, and aspirin in patients with COVID-19^26–30^. Some evidence suggested that the use of DDP-4, metformin, and statin may be associated with better prognosis in COVID-19 patients^27,30,31^. However, none of these effects has been validated yet. Due to the data structure and study design, we were also unable to effectively investigate the independent effects of such drugs. A further investigation is warranted, and several clinical studies including NCT04467931, NCT04510194, NCT04365309, and NCT04407273, are being planned or conducted to examine the potential association of the drugs with prognosis in COVID-19 patients^32^.

Our data had information regarding infection route, which showed that patients at nursing homes had worse prognosis whereas those who contracted the disease from large clusters had better outcomes. This may be attributed to the age distribution and the status of underlying diseases in these groups; most nursing home residents are elderly people with underlying diseases, whereas the infection clusters in Korea during the current outbreak were mostly churches and service call centers where a majority of attendees were young.

We tested several machine learning algorithms because the most appropriate algorithm may differ depending on data structure and a given task. LASSO and linear SVM demonstrated high sensitivities (> 90%) and almost perfect NPV (99.7%) in predicting mortality, which is clinically important because identifying and detecting at-risk patients is more significant than reducing false positive prediction. However, the other models showed low sensitivities despite our efforts to compensate for the class imbalance by up-sampling rare mortality cases and adding class weights when training them. Although we were not able to fully understand and explain this failure to overcome the class imbalance in these models, the difference in variable importance by LASSO and RF may be helpful in explaining the results. The two most important predictors for RF were cluster infection and personal contact where a very small proportion of the patients (0.6%, 42/7256) died, implying that RF chose to focus on detecting negative cases to achieve high AUC. In contrast, LASSO appears to have focused on the predictors associated with increased risk of mortality including old age and DM.

The current study has limitations. First, the data used in this study did not have information regarding laboratory or radiologic results which may also be important prognostic factors^10,17–19,33^. However, our prediction model aims at early prognosis prediction at the time of diagnosis before further diagnostic studies or treatment. Second, the treatments of patients can have a large impact on prognosis; however, we assumed that our patients all had standard therapy. In Korea, all patients are sent to designated hospitals with medical staff and equipment required to provide prompt standard therapy, and this is attributed to aggressive diagnosis and early intervention.

In conclusion, we have developed and validated robust machine learning models, which could be used to predict the prognosis of COVID-19 patients. LASSO and linear SVM demonstrated high sensitivities and specificities for identifying at-risk patients. Age, sex, moderate or severe disability, the presence of symptoms, and comorbidities including hypertension, DM, chronic lung disease or asthma, and cancer were significant risk factors.

## Materials and methods

The Institutional Review Board of National Health Insurance Service Ilsan Hospital (NHIMC 2020-04-026) approved this retrospective Health Insurance Portability and Accountability Act-compliant cohort study and waived the informed consent from the participants, because this study was expected to present no or minimal risk of harm to the participants, and all the data used were anonymized. All methods were performed in accordance with relevant guidelines and regulations.

### Data source

This study used a merged dataset combining relevant information from two data sources provided by the Korean National Health Insurance Service (KNHIS): the database of beneficiaries of national health insurance and the newly added database of patients with confirmed diagnosis of COVID-19. The KNHIS database provides all information regarding reimbursement for outpatient visits and hospital admissions, including sociodemographic information, medical diagnoses, procedures, prescriptions, and national health check-up results. Because virtually all Koreans are covered by national health insurance or Medicaid, the abovementioned information was available for all of our study participants. Detailed information on the KNHIS database including data configuration has been outlined elsewhere^34,35^. Data cannot be shared publicly because of the provisions of the KNHIS which prohibit authors from making the data publicly available and provides a limited portion of anonymized data to researchers for the purpose of a public interest.

### Patients

**Figure 4:**
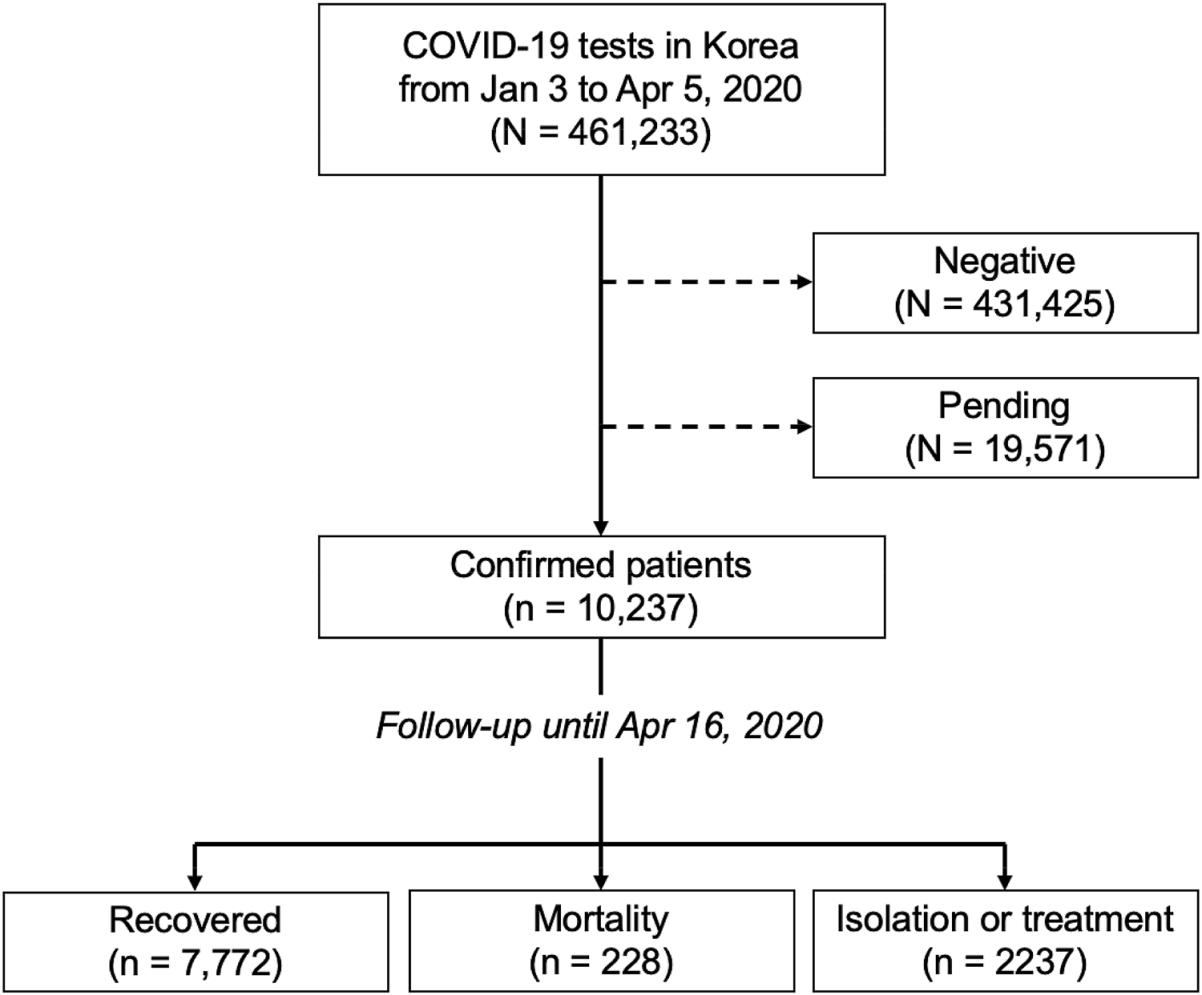
Flow diagram for study participants.

The study included 10,237 Korean patients who had tested positive for COVID-19 from Jan 23, 2020 to Apr 16, 2020 (Fig. 4). Of these patients, 228 (2.2%) had died, 7772 (75.9%) had recovered, and 2237 (21.9%) were still in isolation or being treated. Sixty-seven patients lacked information on their income statuses and were excluded for cox proportional hazard regression and estimation of variable importance by machine learning.

The sociodemographic and medical information included as potential predictive factors were age, sex, income level, place of residence, household type, disability, respiratory symptoms, infection route, underlying medical conditions, and medication (Table 1). The income level was divided into five categories; people in the lowest level were covered by Medicaid, and the four upper levels included quartiles of the insured patients based on the amount of their monthly contributions. Household type had two categories: seniors (> 65 year) living alone and other house types. There were five categories of infection route: personal contact with an infected person or visit to an affected area, cluster infection (e.g., from a religious gathering or packed workplace), infection at a nursing home, infection from abroad, and unclassified. In the KNHIS database, diagnoses were coded according to the Korean Standard Classification of Diseases (KCD) version 6, which is based on the International Classification of Diseases 10th revision (ICD-10). The operational definitions of the medical conditions used in this study are presented in Supplementary Table 2. Based on the prescription information provided by the KNHIS, patients were considered to be on medication if they had received a prescription that lasted more than 180 days for the last year. Medications reported to be potentially associated with COVID-19 prognosis were examined in this study^11,26,30,36,37^: various types of antihypertensive drugs (ACE inhibitors, AR blockers, beta-blockers, calcium channel blockers and loop diuretics), antidiabetic drugs (acarbose, sufonylurea, metformin, and DDP-4), and lipid-lowering drugs (statin and fenofibrate), non-steroidal anti-inflammatory drug, and aspirin.

### Statistical analysis

The Kruskal–Wallis test was performed to compare the age groups. The Cox proportional hazards model was used to assess the hazard ratios of mortality from COVID-19. Following univariable analyses, multivariable analyses were performed to identify independent predictive factors, first with medication usage excluded, and then with underlying disease excluded. This is because the existence of underlying diseases and medication usage are expected to have strong correlations with mortality from COVID-19. Recovered or undetermined cases were censored at the date of recovery or the date of last follow-up (April 16, 2020), respectively. Two-sided probability values of < 0.05 were considered statistically significant. The statistical analyses were performed using the SAS software (version 9.4.3.0; SAS Institute, Cary NC, USA).

### Machine learning

The dataset was randomly split into training and test sets in a ratio of 7:3 while preserving the same proportion of mortality in both sets. Because only a small proportion (2.2%) of the study population died of COVID-19, using accuracy as an evaluation metric is inappropriate. In our study population, an accuracy of 97.8% could be achieved simply by predicting no death every time, which would be a useless classifier as it would result in zero sensitivity. To mitigate the issue of imbalanced classes, we oversampled rare cases, performed class weighting, and used an evaluation metric of AUC that is more appropriate for data with imbalanced classes than accuracy, when fitting our model in the training set.

The machine learning algorithms used in this study were LASSO, linear SVM, RBF-SVM, RF, and KNN. Including irrelevant features in a machine learning model likely results in overfitting and can undermines the generalizability of a prediction model^38^. Thus, LASSO and SVM were regularized using L1-norm which automatically selects important features^39^. For RF, *k* most important features were selected based on the variable importance, with *k* being a hyperparameter that is optimized through cross validation. For KNN, only the features that had been independently associated with mortality in the multivariable Cox regression were used as input data. After the feature selection, the algorithms were trained and tested for classifying mortality vs. recovery after excluding 2237 undetermined cases (i.e., patients who were not cured nor died, but were still in isolation or being treated at the last follow-up). Subsequently, we developed models to predict 14- or 30-day mortality, for which the study population was divided into two groups: patients who died of COVID-19 vs. those who did not within 14 days or 30 days after diagnosis, respectively. Hyperparameter optimization was performed through tenfold cross validation using grid search in the training set. After each optimal model was found, it was fit in the entire training set and tested in the test set.

Using LASSO and RF, the importance of all variables were evaluated and scaled to have a maximum value of 100. Information regarding variable importance was not obtainable from SVM or KNN. Machine learning was performed using the R software (version 3.4.4, R Foundation for Statistical Computing, Vienna, Austria) with the caret package (version 6.0-86).

## Supplementary information

Supplementary Information
