# Supplementary material for: Machine learning prediction for mortality of patients diagnosed with COVID-19: a nationwide Korean cohort study
Source: Sci Rep. 2020 Oct 30;10:18716. doi: 10.1038/s41598-020-75767-2 (PMC7599238; doi:10.1038/s41598-020-75767-2)

Supplementary materials

Title: Machine learning prediction for mortality of patients diagnosed with COVID-19: a nationwide Korean cohort study

Chansik An[1], Hyunsun Lim[1], Dong-Wook Kim[2], Jung Hyun Chang[1,3], Yoon Jung Choi,[1,4*], and Seong Woo Kim[5]

[1]Research Institute, National Health Insurance Service Ilsan Hospital, Goyang, Korea.
[2]Department of Big Data, National Health Insurance Service, Wonju, Korea.
[3]Department of Otolaryngology-Head and Neck Surgery, National Health Insurance Service Ilsan Hospital, Goyang, Korea.
[4]Department of Pathology, National Health Insurance Service Ilsan Hospital, Goyang, Korea.
[5]Department of Physical Medicine and Rehabilitation, National Health Insurance Service Ilsan Hospital, Goyang, Korea.

***Correspondence to:**

Yoon Jung Choi, MD

Department of Pathology, National Health Insurance Service Ilsan Hospital, Goyang, Korea.
Phone: +82-31-900-0891; E-mail: chris316@nhimc.or.kr

***Current address:**
Department of Pathology, Yongin Severance Hospital, Yonsei University College of Medicine, Yongin, Korea.
Phone: +82-31-5189-8447; E-mail: chris316@yuhs.ac

Supplementary Table 1. Optimal hyperparameters

Classifier	Mortality vs. Recovery	Mortality vs. 14-day Survival	Mortality vs. 30-day Survival
LASSO	Lambda = 0.0001	Lambda = 0.003	Lambda = 0.00085
Linear SVM	C = 1	C = 0.5	C = 1
RBF-SVM	Sigma = 0.0198<br>C = 0.5	Sigma = 0.0194<br>C = 1	Sigma = 0.0187<br>C = 1.2
Random Forest	No. of features = 11<br>Mtry = 15	No. of features = 12<br>Mtry = 5	No. of features = 9<br>Mtry = 12
KNN	K = 15	K = 5	K = 5

LASSO, least absolute shrinkage and selection operator; SVM, support vector machine (SVM); RBF, radial basis function kernel; RF, random forest; KNN, k-nearest neighbors.

Supplementary Table 2. Operational definitions of underlying medical conditions

Medical condition	ICD-10 (WHO 2016)
Hypertension	I10, I11, I12, I13, I15
Diabetes mellitus	E10–14
Hyperlipidemia	E78.0–78.5
Cardiovascular disease	I13, I20–25, I48–50, I26.0
Cerebrovascular disease	I60–64, I69 (and two other conditions*)
Cancer	C (and hospital admission)
Lung disease or asthma	J12–18, J41–47, J60–67, J70.1, J70.3, Z99.81
Chronic renal disease	N18, I12, I13, Z49.0–49.2, Z99.2
Mental illness	F
Chronic liver disease	B18, K70.2–70.4, K71.1, K71.3–71.5, K71.7, K72–74, Z94.4

A patient was considered to have an underlying medical condition if she or he had the diagnosis four times or more at least one year during the past five years.

ICD-10, the 10th revision of the International Statistical Classification of Diseases and Related Health Problems; WHO, World Health Organization

* hospital admission and radiologic evidence on computed tomography or magnetic resonance imaging

Supplementary Figure 1. Variable importance by LASSO and Random Forest: Mortal ity vs. Survival within 14 days after diagnosis

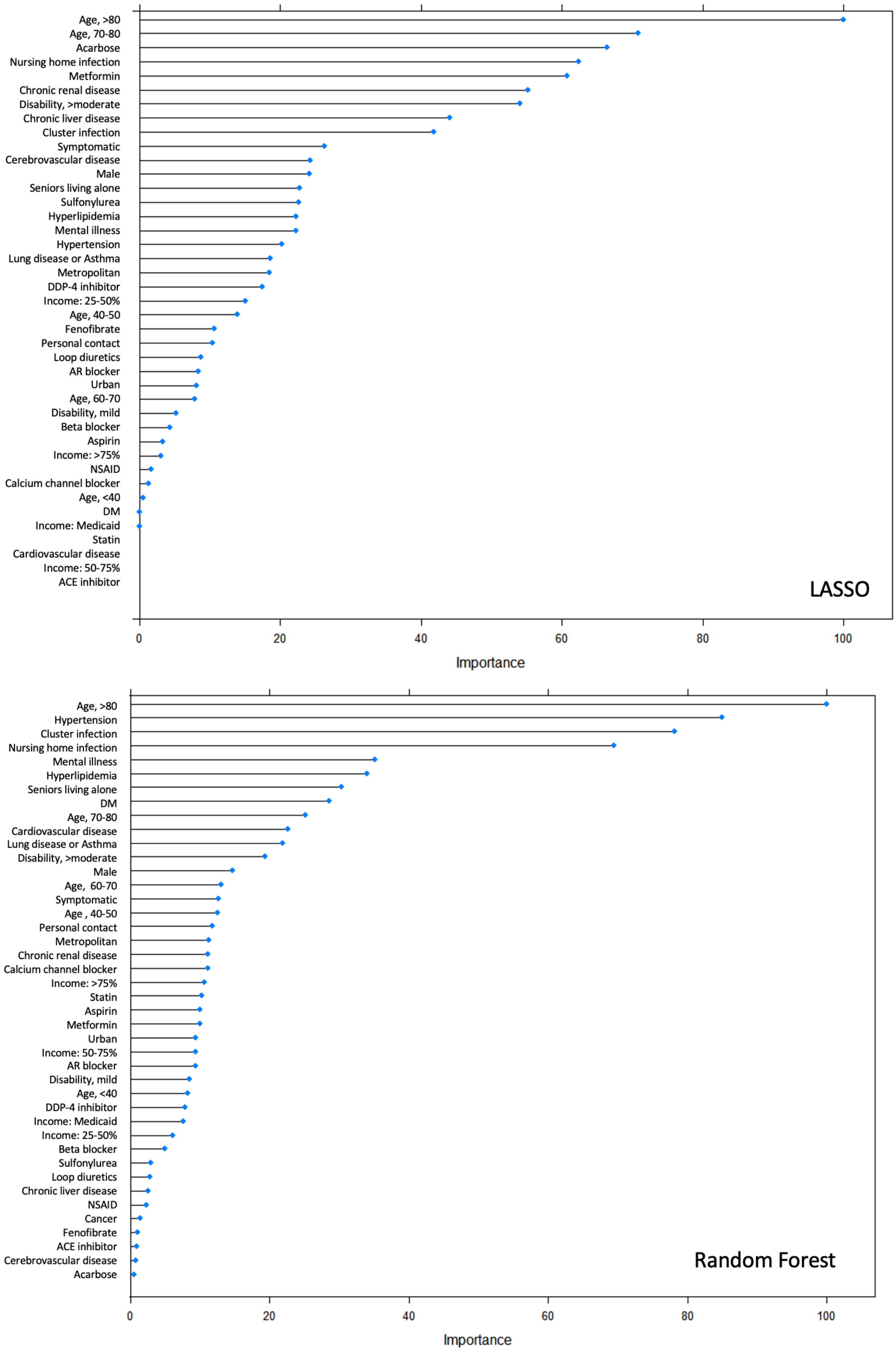

Supplementary Figure 2. Variable importance by LASSO and Random Forest: Mortality vs. Survival within 30 days after diagnosis

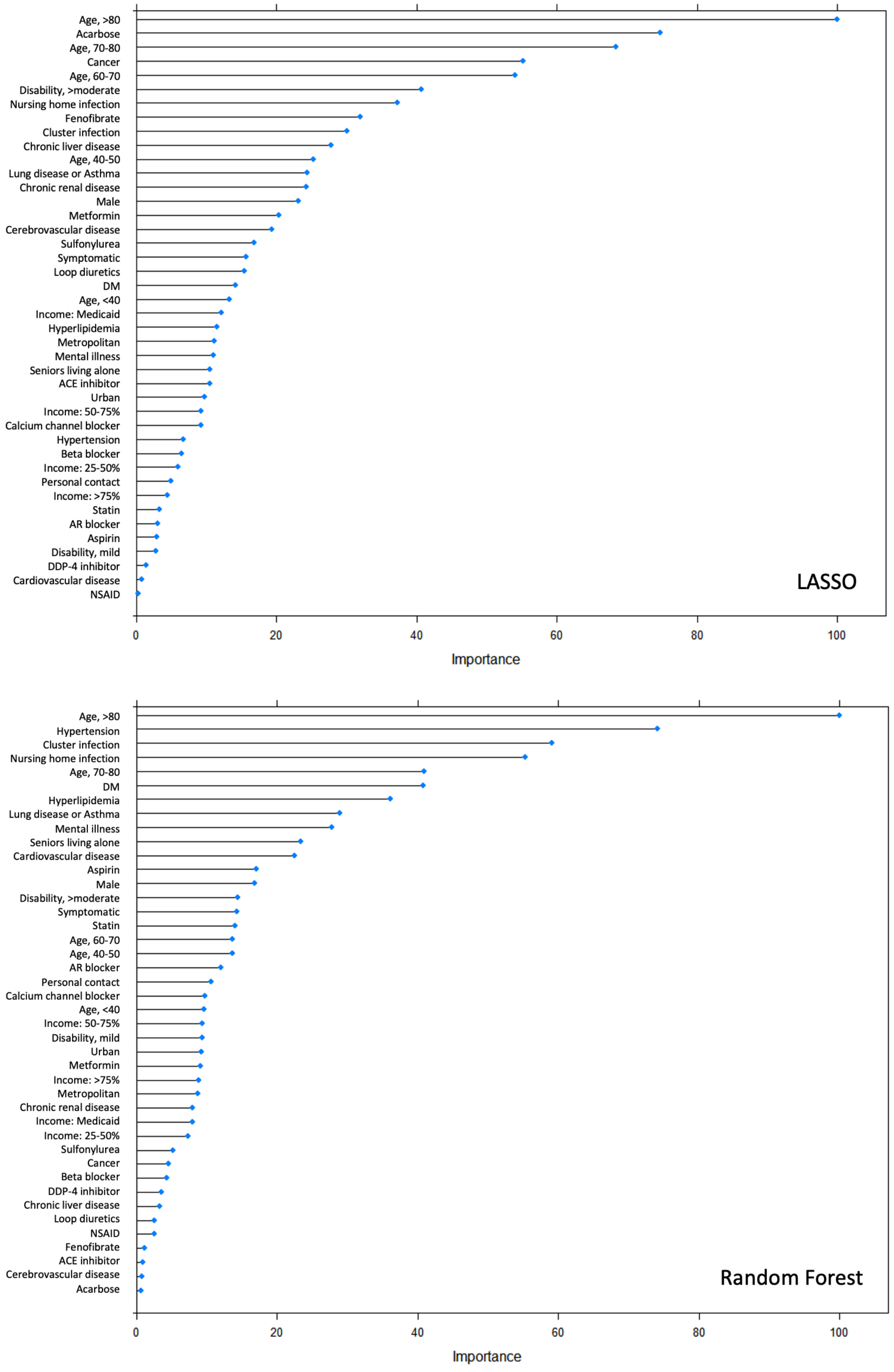

Supplement: Supplementary file 1 — Supplementary Information [file 41598_2020_75767_MOESM1_ESM.pdf]
